# Trends in Cancer Incidence and Potential Associated Factors in China

**DOI:** 10.1001/jamanetworkopen.2024.40381

**Published:** 2024-10-21

**Authors:** Mandi Li, Meijing Hu, Lin Jiang, Jiao Pei, Cairong Zhu

**Affiliations:** 1 Department of Epidemiology and Health Statistics, West China School of Public Health and West China Fourth Hospital, Sichuan University, Chengdu, China; 2 The Second Affiliated Hospital of Kunming Medical University, Kunming, China; 3 Sichuan Cancer Hospital & Institute, Sichuan Cancer Center, School of Medicine, University of Electronic Science and Technology of China, Chengdu, China

## Abstract

**Importance:**

Timely analysis of cancer incidence trends is crucial for cancer prevention and control, which is a public health priority in China.

**Objective:**

To estimate incidence trends for 32 cancers in China from 1983 to 2017 and project changes to 2032, assessing distinct changes due to risk factors and an aging population.

**Design, Setting, and Participants:**

This population-based cohort study used data from the Cancer Incidence in Five Continents database (1983-2017). New cases of 32 cancers were collected. Data were analyzed from October 15, 2023, to May 23, 2024.

**Main Outcomes and Measures:**

Age-standardized incidence rate (AIR) standardized to the World Health Organization World Standard Population, average annual percentage changes (AAPC) using joinpoint regression, and percentage change due to aging and risk factors, using Møller decomposition analysis, stratified by cancer and sex.

**Results:**

A total of 3 677 027 new cancer cases (54.9% male, 45.1% female) were included in the analysis. Increased AIRS were observed for 11 cancers in males and 14 in females from 1983 to 2017, with trends expected to continue until 2032. Thyroid cancer showed the highest increase (AAPC: 7.82% in males; 95% CI, 6.92%-10.38%; 8.59% in females; 95% CI, 7.84%-10.42%), followed by prostate (4.71%; 95% CI, 3.12%-9.95%) and kidney (3.61%; 95% CI, 3.11%-5.82%) cancers in males, and cervical (4.43%; 95% CI, 3.36%-9.44%) and kidney (3.66%; 95% CI, 2.98%-6.86%) cancers in females. The AIRs of lung cancer tended to decrease in males but increase in females during 1983-2017. In contrast, the AIRs of stomach, liver, larynx, and nose and sinuses cancers decreased from 1983 to 2032. From 2018 to 2032, cancer cases were projected to increase primarily due to risk factors for 18 cancers in males and 11 in females, while aging would be associated with the increase in other cancers.

**Conclusions and Relevance:**

In this population-based cohort study of incident cancer in the general population, the cancer landscape in China is evolving, with an increasing incidence primarily due to risk factors in 20 cancers and aging in others. Primary prevention efforts to reduce risk exposure are crucial, and further basic research is needed. Additionally, second prevention efforts are imperative to improve cancer survival, particularly among older individuals.

## Key Points

**Question:**

How have the landscape of cancer incidence and associated factors changed in China from 1983 to 2017?

**Findings:**

In this cohort study of 3 677 027 new cases of 32 cancers from the Cancer Incidence in Five Continents database, age-adjusted incidence rates for most cancers have been increasing, primarily due to risk factors. In contrast, the age-adjusted incidence rates for some cancers are decreasing, while the absolute total number of cases would increase due to an aging population.

**Meaning:**

The results of this study highlight the need to enhance primary prevention efforts to reduce risk exposure and secondary measures to improve cancer survival among older individuals.

## Teaser

This cohort study examines changes in the overall incidence and type of cancer in China between 1983 and 2017 and estimates further changes until 2032.

## Introduction

Cancer is currently the first cause of premature mortality in China, posing a major obstacle to life expectancy improvement.^1^ China accounted for approximately 23.7% of the world’s new cancer cases in 2020 and the incidence rate is expected to continue increasing in the coming decades,^2,3^ making cancer prevention and control a public health priority in China.

Timely and valid analysis of population-based cancer incidence trends is fundamental for cancer prevention and control efforts. However, due to the inherent characteristics of registration data collection and quality control, there is a delay in data release.^4^ Thus, accurate prediction of incidence becomes indispensable to compensate for this data lag^5^ and estimate the future cancer burden. To our knowledge, previous studies on future incidence estimation focused on a maximum of 13 types of cancers at the national level in China,^6^ offering limited insights into long-term patterns of all distinct cancer types. When analyzing the time trend of cancer incidence, it is imperative to explore the potential factors associated with the trends, as this is pivotal for informed policy design. For instance, while interventions have reduced liver and stomach cancer rates in China, an aging population has led to more new cases, indicating a need for targeted intervention efforts for the older population.^7,8^ Conversely, the increase in breast cancer cases is observed to be associated more with risk factors than aging,^9^ highlighting the importance of primary prevention efforts aimed at reducing exposure to modifiable risk factors. The socioeconomic development in China has brought about substantial transformations in both demographic factors (including population aging and growth) and cancer-related risk factors (eg, environmental factors, and lifestyle and behavior of the Chinese population).^10,11,12^ Consequently, it is essential to assess the varying contributions of risk factors alongside the aging population to the development and changes in the incidence of distinct cancer type.

In view of the updated incidence data reported to Cancer Incidence in Five Continents in 2023, we conducted a comprehensive update on the evolving incidence trends of 32 cancers in China since 1983 and extrapolated them into the future until 2032, portraying the shifting cancer incidence spectrum. Additionally, we assessed the association of risk factors and aging population with cancer incidence. We aimed to provide the latest database evidence for cancer prevention and control. The most encompassed distinct cancer types enhanced our ability to assess the overall cancer burden and identify predominant cancers, facilitating a more precise allocation of priority health resources by the government.

## Methods

### Data Source and Population

Our study was based on publicly available, deidentified data and, therefore, the National Health Commission of the People’s Republic of China determined it exempt from ethical and informed consent. This study followed the Strengthening the Reporting of Observational Studies in Epidemiology (STROBE) reporting guideline.

Cancer incidence was collected from the Cancer Incidence in Five Continents based on the cancer and registry dictionary.^13,14,15,16,17,18,19^ Registries in the mainland of China with 5-year period data in each volume were selected. A total of 3 677 027 new cases of 32 cancers were collected from volumes VI to XII (1983-1987 to 2013-2017), including 2 018 339 males (54.9%) and 1 658 688 females (45.1%). Population data were obtained from the United Nations Department of Economic and Social Affairs Population Division, 2022 Revision of World Population Prospects.^20^

### Statistical Analysis

Age-adjusted incidence rate (AIR) was standardized to the World Health Organization World Standard Population,^21^ using the direct method,^22^ by sex and cancer. The log-linear joinpoint regression model was applied to analyze the temporal trends from 1983 to 2017. The joinpoint regression model composed of a few continuous linear phases is often useful to describe changes in trend data, determining joinpoints and obtaining the annual percentage change (APC) and average annual percentage change (AAPC) with 95% CI.^23^ For analysis of cancer mortality and incidence data,

(*x_1,_ γ_1_*)…(*x_n,_ γ_n_*), *x_1_*<<…<<*x_n_*

a log-linear model is often used:

E[y|x] = e^β0 + β1 x+ δ1 (x−τ1) + +…+ δk (x−τk)+^

where the τ*_k_* values are the unknown joinpoints and *a^+^* = *a* for *a* >0 and 0 otherwise. The APC and AAPC represent a 5-year percentage change and a 5-year average percentage change in this study, as the origin data were displayed every 5 years.

To project the incidence from 2018 to 2032, we used bayesian age-period-cohort (BAPC) analysis with integrated nested Laplace approximations.^24^ In the BAPC model, all unknown parameters are treated as random with appropriate prior distributions. It can be written as log(λ_ij_) = *μ* + *α_i_* + *β*_j _+ *γ_k_* + *z_ij_*. Here, *λ_ij_* denotes the incidence rate for each age group (i) and period (j); *μ* represents the intercept, and *α_i_* denotes age, *β_j_* denotes period, and *γ_k_* denotes cohort effects; *z_ij_* denotes parameters for additional unstructured heterogeneity, for which the mean 0 gaussian distribution

*z_ij_∼Ν(0,k_z_^−1^)*

was used, where *k_z_* ^−1^ denotes the precision parameters. For all the precision parameters, the hyperprior of γ distributions *k ∼ G*(*a,b*) were assumed with shape parameter *a* = 1 and rate parameter *b* = 0.005. For age, period, and cohort effect, a random walk of first order and second order is commonly used due to the expectation that effects adjacent in time might be similar.^24,25^ The random walk of second-order prior assumes a linear time trend, and the outcomes are predicted from its 2 immediate predecessors by linear extrapolation:

*β_j_|β_1,…,_β_j −1,_ k_β_ ∼ N* (*2β_j −1_* – *β_j −2,_ k_β_*
^−1^)

for *j* greater than or equal to 3; the random walk of first order prior assuming a constant trend over the time scale:

(*β_j_|β_1,…,_β_j −1,_k_β_ ∼ N (β_j −1,_ k_β_^−1^*)

for *j* greater than or equal to 2.^24,25^ In our study, the random walk of first-order priors are given priority. The final model was determined based on the performance of our retrospective projection of incidence in 2013-2017, evaluated using 3 metrics (eMethods in Supplement 1). We also conducted a sensitivity analysis of projections using alternative hyperprior values, such as *a* = 1 and *b =* 0.0005, to enhance the robustness and credibility of the results (eTable 5 in Supplement 1).

The decomposition analysis of changes in cancer incidence proposed by Møller et al^26^ was conducted to explore the factors associated with the changes due to 3 explanatory components: risk factors, aging population, and population growth in China.^26^ Let *N_ras_* be the number of cases given a cancer risk *r*, an age structure *a* and a population size *s*, where *r*, *a*, and *s* are levels for the observed period 2013-2017 (*o*) or the future period, ie, 2018–2022 (*f*). First, we estimated the expected number of cases in 2028-2032 by multiplying the number of individuals in the population in 2028-2032, age-specific incidence rate in 2013-2017, and the age structure in 2013-2017 (*N_oof_*) and 2028-2032 (*N_off_*). Then, we estimated the change due to population growth by subtracting actual cases in 2013-2017 (*N_ooo_*) from *N_oof_*, that is (*N_oof_ – N_ooo_*), the change due to population aging (*Δ_age_*) by calculating the differences between *N_off_* and *N_oof_*, that is (*N_off_* − *N_oof_*), and the change due to risk factors (*Δ_risk_*) by subtracting *N_off_* from 2020 actual cases (*N_fff_*), that is (*N_fff_ – N_off_*). Third, we calculated the difference as a percentage: *Δ_risk_*/*N_ooo_*, the changes due to change of risk factors, and *Δ_age_/N_ooo_*_,_ the changes due to aging population. All analyses were performed using SAS, version 9.4 (SAS Institute LLC), Joinpoint Regression Program, version 4.9.0.0 (IMS Inc), and R, version 4.0.2 (R Foundation for Statistical Computing). Statistical significance was set at a 2-tailed threshold of *P* < .05 throughout the study.

## Results

### Cancer Incidence During 1983-2017

The age-adjusted incidence rates of top cancers in males and females from 1983 to 2017 are displayed in Table 1. For all cancers combined, the crude incidence increased from 236.24 in 1983-1987 to 367.57 per 100 000 person-years in 2013-2017 among males (AAPC = 1.62%; 95% CI, 1.41%-1.99%) and 176.17 to 314.39 per 100 000 person-years among females (AAPC = 2.01%; 95% CI, 1.70%-2.72%) (Table 2). The standardized incidence in males fluctuated between 193.21 and 219.22 per 100 000 person-years, while in females it increased from 142.94 to 183.81 per 100 000 person-years, with an AAPC of 0.99% (95% CI, 0.83%-1.35%).

**Table 1.** Age-Adjusted Incidence per 100 000 Person-Years by Sex for Top Cancers in China, 1983-2017

Cancer type by *ICD-10* code	Age-adjusted incidence per 100 000 person-years						
	1983-1987	1988-1992	1993-1997	1998-2002	2003-2007	2008-2012	2013-2017
C33-34, lung (including trachea and bronchus)	34.39	37.23	34.61	33.83	31.68	33.05	37.15
Female	21.32	22.53	21.94	20.11	20.05	21.64	25.44
Male	49.40	54.46	47.91	50.13	44.49	45.30	49.63
C18-21, colorectum	14.03	16.29	15.84	22.98	19.91	20.31	20.04
Female	13.39	15.21	15.08	21.16	18.07	17.56	16.40
Male	14.81	17.79	16.68	25.33	22.03	23.25	23.86
C16, stomach	31.90	29.04	23.74	22.23	20.01	19.21	19.11
Female	19.34	18.42	15.14	15.00	13.19	12.15	11.52
Male	46.49	41.56	32.82	30.83	27.40	26.73	27.08
C22, liver	22.27	20.18	18.25	17.22	17.00	15.83	16.25
Female	11.42	10.39	9.71	8.32	8.56	7.99	8.12
Male	33.67	30.50	27.04	26.66	25.65	23.83	24.53
C50, breast (female)	20.17	24.50	23.31	31.34	32.71	34.46	32.79
C73, thyroid	1.36	1.64	1.71	2.62	3.63	8.03	15.17
Female	1.94	2.42	2.59	4.00	5.66	12.21	22.94
Male	0.79	0.89	0.83	1.28	1.66	3.91	7.47
C15, esophagus	10.47	8.61	10.22	6.53	10.04	9.55	10.74
Female	6.63	5.16	6.74	2.88	6.20	5.37	5.66
Male	15.12	12.81	13.90	10.70	14.15	13.93	16.02
C25, pancreas	4.78	4.99	4.83	5.87	5.02	5.20	5.16
Female	3.86	4.00	4.32	4.86	4.32	4.38	4.26
Male	5.87	6.12	5.37	7.01	5.78	6.06	6.09
C61, prostate	1.49	2.02	2.54	5.93	7.25	8.01	9.12
C53, cervix uteri	5.37	3.49	2.56	2.90	5.79	9.33	10.89
C00-96, all sites	176.28	179.04	166.99	187.07	180.50	187.29	198.74
Female	142.94	148.00	142.38	162.46	162.96	172.93	183.81
Male	215.77	217.37	193.21	219.22	201.90	204.39	216.17

Abbreviation: *ICD-10*, *International Statistical Classification of Diseases and Related Health Problems, Tenth Revision.*

**Table 2.** Trends in Cancer Incidence per 100 000 Person-Years by 32 Cancer Types in China for 1983-2017

Cancer type by *ICD-10* code	AAPC (95% CI)^a^			
	Crude rate		World AIR	
	Male	Female	Male	Female
C00-14, oral cavity and pharynx	1.87 (0.81 to 4.29)	0.83 (−0.05 to 2.75)	0.74 (−0.37 to 3.50)	−0.11 (−1.37 to 2.69)
C15, esophagus	2.34 (1.85 to 3.85)	1.32 (0.68 to 2.87)	0.73 (0.07 to 2.71)	−0.28 (−1.02 to 1.78)
C16, stomach	−0.28 (−0.61 to 0.48)	−0.36 (−0.66 to 0.08)	−1.74 (−1.94 to −1.43)	−1.67 (−1.96 to −1.07)
C17, small intestine	3.60 (2.98 to 5.17)	3.03 (1.87 to 6.23)	2.21 (1.66 to 3.47)	1.76 (0.69 to 4.59)
C18-21, colorectum	2.72 (1.98 to 4.44)	1.50 (0.47 to 3.63)	1.27 (0.75 to 2.34)	0.84 (0.50 to 1.63)
C22, liver	0.23 (0.19 to 0.30)	0.25 (0.18 to 0.46)	−0.83 (−1.17 to −0.01)	−1.18 (−1.36 to −0.77)
C23-24, gallbladder, etc	2.74 (2.34 to 3.53)	2.27 (1.61 to 3.76)	0.27 (−0.43 to 1.43)	−0.43 (−1.68 to 2.10)
C25, pancreas	1.75 (1.36 to 2.56)	1.62 (1.09 to 2.63)	0.15 (−0.19 to 0.76)	0.36 (0.17 to 0.66)
C30-31, nose, sinuses, etc	−0.99 (−1.34 to −0.41)	−1.16 (−1.96 to 0.40)	−2.04 (−2.36 to −1.53)	−2.18 (−3.09 to −0.45)
C32, larynx	0.36 (−0.33 to 1.74)	−3.93 (−4.70 to −2.87)	−1.05 (−1.46 to −0.43)	−5.29 (−6.62 to −3.43)
C33-34, lung (including trachea and bronchus)	1.52 (1.26 to 2.18)	1.80 (1.61 to 2.08)	−0.01 (−0.44 to 1.13)	0.37 (0.12 to 0.82)
C40-41, bone	−0.31 (−0.49 to 0.01)	−0.30 (−0.64 to 0.47)	−1.16 (−1.26 to −1.05)	−1.03 (−1.46 to −0.14)
C43, melanoma of skin	2.12 (1.53 to 3.52)	2.50 (2.22 to 3.13)	0.76 (0.39 to 1.64)	1.54 (1.19 to 2.44)
C44, other skin	3.47 (2.90 to 4.89)	4.38 (3.77 to 5.81)	1.84 (1.11 to 3.80)	2.70 (2.15 to 4.50)
C47, C49, connective tissue	−0.18 (−2.03 to 4.01)	−0.24 (−2.23 to 4.02)	−1.08 (−2.9 to 2.44)	−0.75 (−2.68 to 2.77)
C50, breast	1.26 (−0.01 to 4.43)	2.72 (2.36 to 4.34)	−0.11 (−1.07 to 1.98)	1.26 (0.33 to 3.45)
C53, cervix uteri	NA	4.97 (3.89 to 9.93)	NA	4.43 (3.36 to 9.44)
C54, corpus uteri	NA	3.15 (2.01 to 5.87)	NA	2.20 (1.23 to 4.37)
C56, ovary	NA	1.86 (1.54 to 2.68)	NA	0.94 (0.67 to 1.62)
C60, penis	2.99 (2.19 to 5.35)	NA	0.59 (0.08 to 2.82)	NA
C61, prostate	8.79 (8.21 to 12.08)	NA	4.71 (3.12 to 9.95)	NA
C62, testis	−0.85 (−1.79 to 0.93)	NA	−0.91 (−2.12 to 1.47)	NA
C67, bladder	2.01 (1.66 to 3.24)	0.92 (−0.55 to 3.94)	0.24 (−0.03 to 1.18)	−0.51 (−1.82 to 2.00)
C64-66, C68 kidney, etc	5.16 (4.43 to 8.06)	5.12 (4.24 to 8.75)	3.61 (3.11 to 5.82)	3.66 (2.98 to 6.86)
C69, eye	−0.82 (−1.83 to 1.25)	0.00 (−1.21 to 2.62)	−1.34 (−2.74 to 1.22)	−0.25 (−0.96 to 0.71)
C70-72, brain, central nervous system	0.77 (0.35 to 1.48)	1.09 (0.71 to 2.02)	−0.12 (−0.43 to 0.55)	0.06 (−0.31 to 1.23)
C73, thyroid	8.51 (7.48 to 11.49)	9.14 (8.41 to 11.22)	7.82 (6.92 to 10.38)	8.59 (7.84 to 10.42)
C74-75, other endocrine	0.32 (−0.63 to 2.49)	−1.81 (−2.93 to 1.02)	−0.30 (−1.07 to 1.25)	−2.14 (−3.62 to 2.17)
C81, Hodgkin lymphoma	0.74 (0.23 to 1.81)	0.32 (−0.41 to 1.64)	0.04 (−0.64 to 1.23)	0.08 (−0.77 to 1.56)
C82-86, C96, non-Hodgkin lymphoma	1.57 (0.91 to 3.03)	2.03 (0.97 to 4.48)	0.31 (−0.24 to 1.38)	1.26 (1.03 to 1.98)
C88, C90, multiple myeloma	3.62 (2.51 to 6.99)	3.90 (3.03 to 6.47)	2.14 (1.50 to 3.6)	2.69 (2.06 to 4.57)
C91-95, leukemia	1.41 (1.14 to 2.13)	1.22 (0.93 to 1.83)	0.64 (0.29 to 1.48)	0.19 (−0.08 to 0.63)
C00-96, all sites	1.62 (1.41 to 1.99)	2.01 (1.70 to 2.72)	0.17 (−0.09 to 0.62)	0.99 (0.83 to 1.35)

Abbreviations: AAPC, 5-year average annual percentage change; AIR, age-adjusted incidence rate; *ICD-10*, *International Statistical Classification of Diseases and Related Health Problems, Tenth Revision*; NA, not applicable.

### Incidence Trends From 1983 to 2032

For 7 of 32 cancer types in males and females (including thyroid, kidney, colorectal, and small intestine cancer; multiple myeloma; and melanoma of the skin), 4 cancer types in males (leukemia, prostate, esophageal, and penis cancer), and 7 cancer types in females (cervical, corpus uteri, breast, ovary, lung, and pancreas cancer; and non-Hodgkin lymphoma), the AIRs showed significantly increasing trends from 1983-1987 to 2013-2017 (Table 2). Among these 18 cancers, thyroid cancer exhibited the greatest increase in AIR for both sexes, with an AAPC of 7.82% (95% CI, 6.92%-10.38%) for males and 8.59% (95% CI, 7.84%-10.42%) for females, followed by prostate (AAPC = 4.71%; 95% CI, 3.12%-9.95%) and kidney (AAPC = 3.61%; 95% CI, 3.11%-5.82%) cancer in males, and cervical (AAPC = 4.43%; 95% CI, 3.36%-9.44%) and kidney (AAPC = 3.66%; 95% CI, 2.98%-6.86%) cancer in females. During 2018-2022 to 2028-2032, the increasing trends of these 18 cancers were estimated to continue (eFigure 2 in Supplement 1). Among males, thyroid cancer was expected to continue to show the most pronounced increase, with the AIR rising 5.28 times from 7.47 in 2013-2017 to 39.42 per 100 000 person-years in 2028-2032, followed by kidney cancer (1.87 times) and prostate cancer (1.82 times). Among females, thyroid cancer would also still show the greatest increase in AIR (4.77 times), followed by cervical cancer (4.05 times) and lung cancer (2.00 times) (eTable 4 in Supplement 1).

For 5 of 32 cancers (stomach, liver, larynx, bone, and nose and sinuses), the AIRs significantly decreased in both females and males from 1983-1987 to 2013-2017 (Table 2). For instance, in males, AIRS decreased from 46.49 per 100 000 person-years to 27.08 (AAPC = −1.74%; 95% CI, −1.94% to −1.43%) for stomach cancer, and from 33.67 per 100 000 person-years to 24.53 (AAPC = −0.83%; 95% CI, −1.17% to −0.01%) for liver cancer. In females, these rates decreased from 19.34 per 100 000 person-years to 11.52 (AAPC = −1.67%; 95% CI, −1.96% to −1.07%) for stomach cancer and from 11.42 per 100 000 person-years to 8.12 (AAPC = −1.18%; 95% CI, −1.36% to −0.77%) for liver cancer. During 2018-2022 to 2028-2032, the AIRs of larynx, liver, stomach, and nose and sinuses cancers were expected to keep decreasing (eFigure 2 in Supplement 1).

### Changes Due to Risk and Population From 2013 to 2032

**Figure 1.**
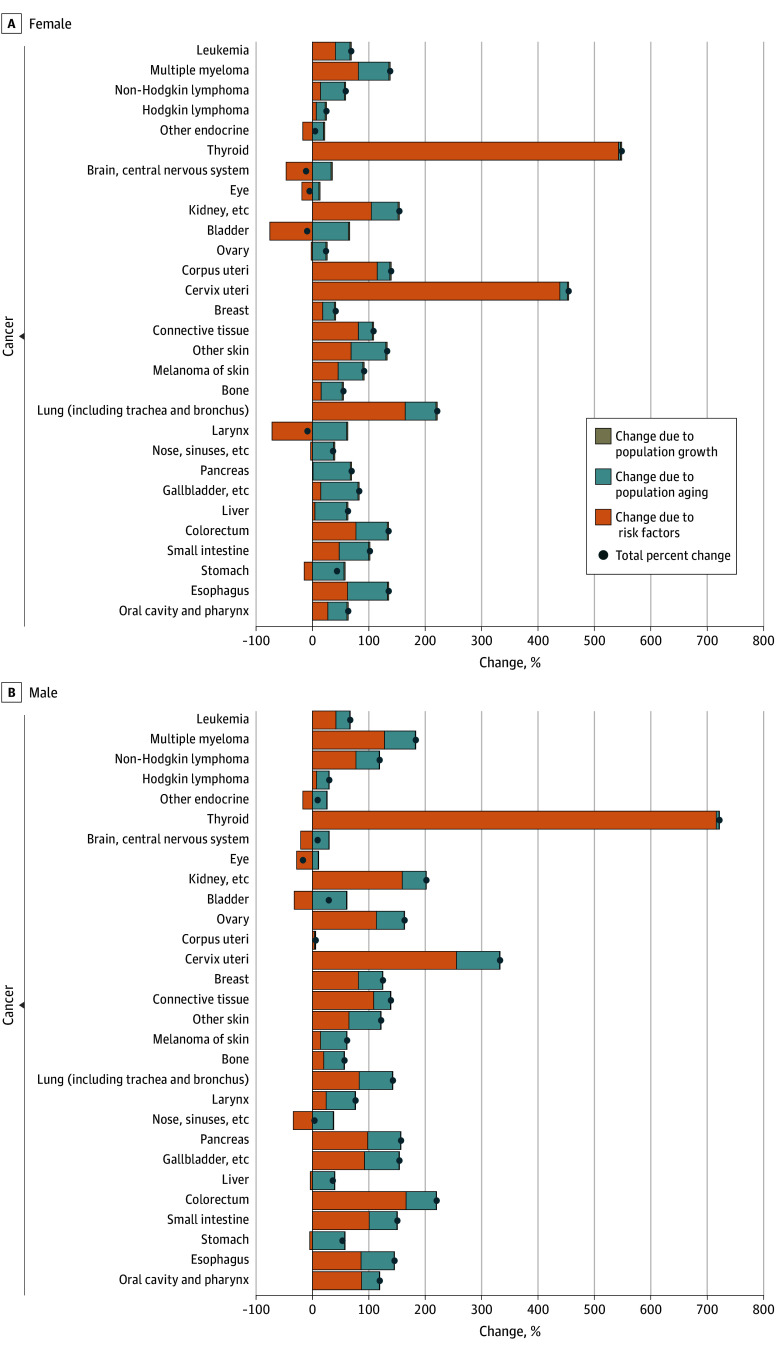
Potential Factors of Changes in New Cases for 32 Cancers in China, 2028-2032 The change was compared with incidence estimated in 2013-2017.

The projection analysis indicated an increase in the number of new cases of cancer from 2018-2022 to 2028-2032 (eTable 4 in Supplement 1). According to the decomposition analysis of the changes in cases by cancer types, for 18 cancers in males (including thyroid, prostate, colorectal, kidney, small intestine, pancreas, esophageal, breast, and lung) and 11 cancers in females (including thyroid, cervical, lung, corpus uteri, kidney, and colorectal), the contribution of risk factors exceeds that of aging (Figure 1). Notably, for thyroid, cervical, and prostate cancer, the increase is primarily attributed to changes in risk factors. Compared with 2013-2017, the percentage changes associated with risk factors in 2028-2032 for thyroid cancer are 721.76% in males and 548.43% in females, and 454.36% for cervical cancer and 332.69% for prostate cancer (Figure 1; eTable 4 in Supplement 1).

Despite the important role of risk factors in the increased cases, for certain cancers, such as liver, stomach, and bladder in males, and stomach and ovary in females, the increase would be entirely due to demographic factors, especially the aging population. Specifically, the aging population would result in a change in stomach cancer of 57.18% in males and 55.84% in females in 2028-2032, compared with 2013-2017 and a 39.11% of change for liver cancer in males. In contrast, the percentage change for stomach cancer due to risk factors would be −4.74% in males and −14.56% in females and −3.82% for liver cancer in males. In fact, the aging population would contribute to the increase in new cases for all 32 cancers by 2028-2032 (percentage change due to aging population was >0), and for 11 cancers in males (including stomach, liver, bladder, bone, eye, brain, and larynx cancer) and 18 cancers in females (including stomach, liver, ovary, pancreas, bone, breast, small intestine, and esophageal). The contribution of the aging population would be greater than that of risk factors (Figure 1; eTable 4 in Supplement 1).

### Cancer Spectrum Changes From 1983 to 2032

**Figure 2.**
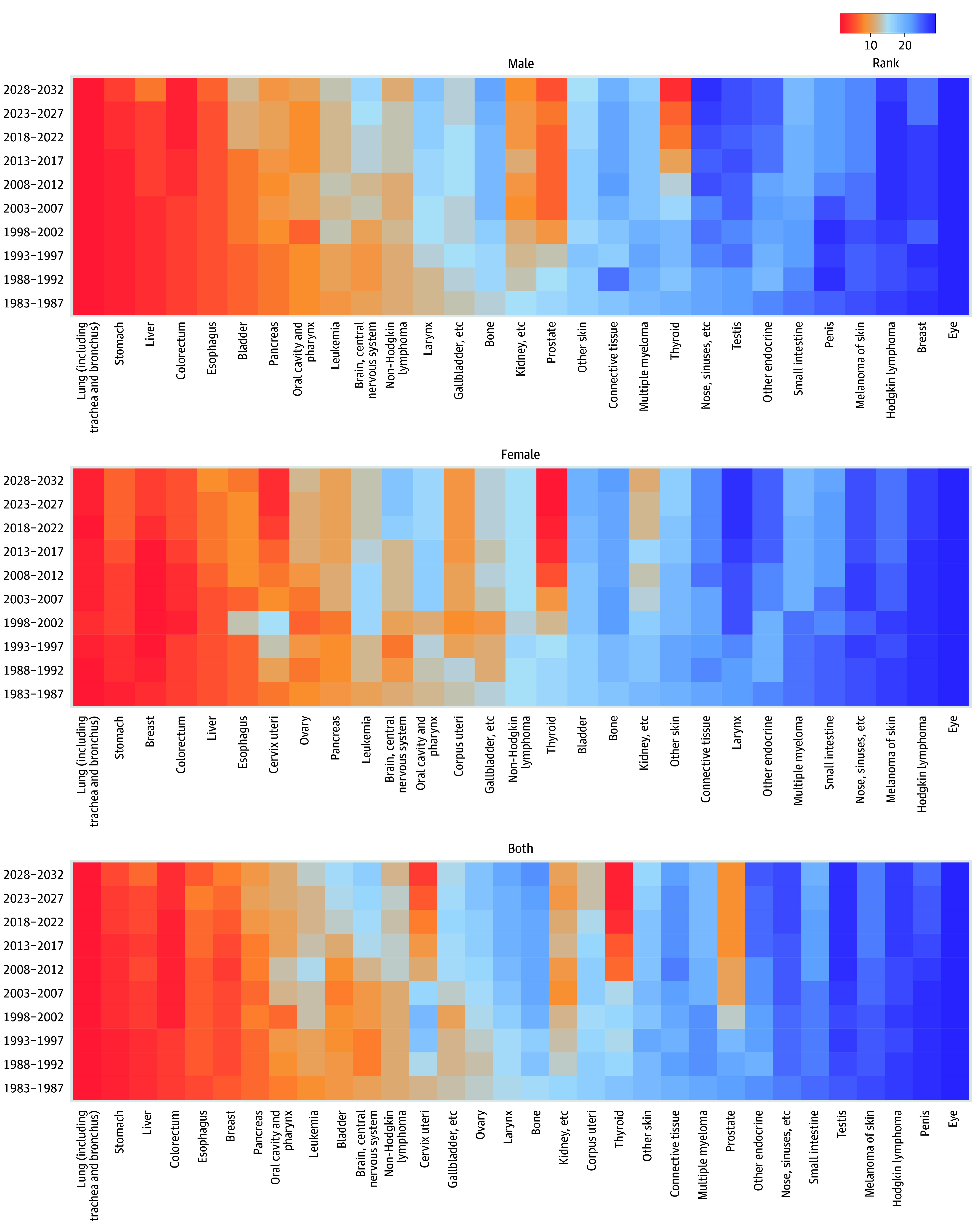
Cancers Ranked by Number of New Cases in China, 1983-2032

For females, lung, stomach, breast, colorectal, and liver cancer were the top 5 leading cancers in 1983-1987. A gradual decrease was observed to fifth most common cancer for stomach cancer and to seventh for liver cancer in 2013-2017, while breast cancer increased to replace lung cancer as the most commonly diagnosed cancer. Thyroid cancer moved from sixteenth position to become the third most common cancer. Cervical cancer also showed an increasing trend in the ranking after 2003-2007 to become the sixth common cancer in 2013-2017. Beyond 2017, liver, stomach, and breast cancer may decrease in the ranking, while thyroid and cervical cancer would continue increasing. Thyroid, lung, cervical, breast, and colorectal cancer are projected to be the top 5 leading cancers in China by 2028-2032 (Figure 2).

For males, lung, stomach, liver, colorectal, and esophageal cancers remained the top 5 most common cancers during 1983-2017. Prostate, thyroid, and kidney cancer all increased in the ranking to become the top 10 most common cancers in 2013-2017. Beyond 2017, thyroid, prostate, and kidney cancer would display a substantial increase in the ranking, while liver, stomach, and esophageal cancer gradually decrease. Lung, colorectal, thyroid, stomach, and prostate cancer are projected to be the top 5 leading cancers in China by 2028-2032. Similar to the whole population, lung cancer remains the most common cancer among males (Figure 2).

## Discussion

This study analyzed cancer incidence trends in China from 1983 to 2032 for 32 cancer types reported to the Cancer Incidence in Five Continents. We found major increases in the AIRs for certain cancers, largely attributed to changing risk factors. Although the AIRs of stomach and liver cancer are decreasing, their burden remains high due to an increasing number of cases observed with population aging. The cancer incidence spectrum is evolving, with thyroid and cervical cancer in females and thyroid and prostate cancer in males potentially becoming more predominant by 2032. However, lung and colorectal cancer remain major disease types, with female lung cancer expected to increase more rapidly than in males.

The incidence rates of most cancers are increasing and would further increase by 2032 in China, with changing risk factors being the primary factors. Although the application of advanced diagnostic technology may lead to overdiagnosis and artificially increase the incidence of certain cancers, the impact should be short term and limited.^27,28^ It is essential to focus on the real causes of cancer. For instance, thyroid cancer has increased sharply in China and is associated with factors such as radiation exposure, obesity, induced abortion, and endocrine-disrupting chemicals from environmental pollution.^27^ Kidney cancer is associated with obesity, smoking, and hypertension,^29^ while prostate cancer risk may be linked to smoking, chemical and pesticide exposure, and low physical activity,^30^ although further studies are needed to confirm this association in China. In terms of cervical cancer, the increase in human papillomavirus infection, smoking rate, and changing sexual behaviors are possible explanations.^31^ For lung cancer, smoking was the most common risk factor, followed by air pollution.^32^ Although lung cancer AIR is higher in males, the increase among females (with much lower smoking rate) is more pronounced, consistent with other studies.^32,33^ This phenomenon may be associated with the faster increase in incidence among younger females compared with younger males in mainland China.^34^ Higher rates in younger females might be attributed to secondhand smoke and household air pollution, particularly among female nonsmokers,^32,33,35,36^ alongside increasing smoking rates among young females.^37^ Additionally, the association between air pollution and lung cancer risk appears stronger in females.^38^ However, strengthening tobacco control policies remains crucial, as evidenced by a marked decrease in lung cancer incidence when we included data from Hong Kong,^39^ which implemented earlier and stricter tobacco controls, compared with the insignificant decrease in mainland China. As for gastrointestinal cancers, the high incidence in China can be attributed to factors such as smoking, low fruit intake, alcohol consumption, and excess body weight.^10,40,41^ Hence, primary prevention through reducing exposure to modifiable risk factors is vital for curbing the substantial and growing cancer burden in China. Further population-based epidemiologic studies are warranted to identify modifiable risk factors, facilitating more effective prevention and control efforts. Furthermore, given that some Western countries have witnessed a decrease in the incidence of certain cancers,^42,43^ it is advisable to draw on their successful prevention measures to formulate suitable policies complying with the evolving situation in China, ultimately reducing the cancer burden on society.^10^

Despite the severity of the increasing incidence of many cancers in China, the fact of a decrease in stomach and liver cancer due to risk factor control, such as controlling *Helicobacter pylori* infection^44^ and hepatitis B virus vaccination,^45^ is still worthy of recognition. In addition, it is reasonable to anticipate that with the reinforcement of the prevention and control measures, these rates are expected to continue decreasing, aligning with our estimations. However, major challenges persist, as *H pylori* infection still affects approximately half of adults^44^ and the prevalence of hepatitis C virus, another critical factor in liver cancer, is increasing.^46^ To further control the burden of stomach and liver cancer, it is imperative to reinforce infection control, enhance early screening and treatment, improve cancer literacy among the population,^41^ and take proactive measures to deal with the impact of aging to improve cancer survival.

Even with decreasing AIRs, the persistent growth and sustained increased levels of liver and stomach cancer cases in China underscore the major role of the aging population. Our results suggest that while prevention and treatment measures have been associated with incidence decrease, they cannot fully offset the influence of aging, which remains the key factor in increased cancer cases. Indeed, the aging population was expected to be associated with a decrease not only in liver and stomach cancers, but also in other cancers, partially, if not entirely. Consequently, efforts to alleviate the cancer burden associated with the aging population should focus on reducing exposure to modifiable risk factors in the younger population, as well as early disease detection and treatment for older individuals, to improve cancer survival.^47^ Furthermore, integrating healthy aging strategies into cancer prevention and control initiatives is imperative.^42^ However, it is essential to emphasize that, while the aging population substantially impacts cancer incidence, primary and secondary prevention strategies remain paramount, as a substantial proportion of cancers are preventable, and such prevention measures are cost-effective, particularly in low- and middle-income countries.^48^

The cancer incidence spectrum in China is transitioning to that of high-income countries.^43^ Our study suggests that colorectal, breast, prostate, and thyroid cancers have gradually become the leading cancers in China, similar to the US, where prostate, colorectal, and breast cancers dominate.^43^ Meanwhile, stomach and liver cancer, which previously ranked higher in China but lower in Western countries, are now decreasing.^43^ However, the cancer spectrum in China retains some characteristics of low-income countries. For instance, cervical cancer rates are increasing and may even surpass breast cancer, which is uncommon in high-income countries.^4^ The complex shift in the cancer spectrum presents considerable challenges for cancer prevention and control in China. First, it is important to be cautious of overdiagnosis in cases of prostate and thyroid cancer. Second, screening coverage for breast, colorectal, and cervical cancers remains insufficient, and public awareness of screening needs improvement.^41,43,49,50^ Given the success of early screening in reducing cervical and colorectal cancer in some Western countries,^51,52^ the Chinese government should enhance policies to promote cost-effective screening among high-risk groups and improve participation rates.^41^ Third, we need to strengthen vaccination efforts, particularly for human papillomavirus, as human papillomavirus vaccination coverage remains low in China.^53^ Additionally, the screening rate among women aged 20 to 64 years is only 25.7%.^49^ Despite the transition of the cancer spectrum in China, lung cancer has consistently been the most common, and is associated with high smoking rates and air pollution linked to economic development.^10^ Therefore, the Chinese government should further intensify antitobacco measures and control both outdoor and indoor air pollution, while researchers should vigorously promote basic research on lung cancer.

This study presents a comprehensive depiction of the current cancer profiles in China, offering insights into potential future trends based on the latest incidence data encompassing a wide range of cancer types, which ensures the provision of up-to-date evidence crucial for guiding effective strategies in cancer prevention and control. Through a retrospective analysis evaluating performance of BAPC models, we derived final models (eMethods in Supplement 1), thus supporting the reliability of our estimations regarding cancer incidence from 2018 to 2032. Additionally, this study specifically examined the contributions of risk factors and demographic structure to the shifts of cancer incidence, providing valuable insights for governments in formulating targeted health policies.

### Limitations

Our study faces certain limitations. First, we were unable to conduct analyses by cancer subtype due to incomplete cancer pathologic classification. Second, the dynamic changes in cancer profiles across rural and urban China cannot be further analyzed due to the data limitations. Nonetheless, we anticipate that ongoing improvements in cancer registration will yield more detailed incidence data, thereby facilitating a more in-depth analysis of characterization of cancer incidence patterns in China.

## Conclusions

In this population-based cohort study, the landscape of cancer incidence in China shifted significantly, resembling that of high-income countries while retaining some low-income country characteristics. Cancers such as thyroid, prostate, colorectal, and cervical cancers showed marked increases and are expected to become more prevalent due to changing risk factors. Although stomach and liver cancer incidence decreased, new cases continue to increase due to an aging population. Despite these shifts, lung cancer remains the most predominant. Addressing increasing cancer rates requires prioritizing primary interventions to reduce risk exposure, strengthening secondary prevention with a focus on early screening and treatment for the older population, and advancing basic research for effective prevention and control.

## Notes

Supplement 1. **eMethods.** Construction of BAPC Models of 32 Cancer Types **eTable 1.** Established BAPC Models for 32 Cancer Types by Sexes **eFigure 1.** Retrospective Projections of Age-Adjusted Incidence Rates of 32 Cancer Types in China for Males and Females **eTable 2.** The Bias and Precision of the BAPC Model for 32 Cancer Types by Sexes **eTable 3.** The Percentage (%) of Observed Age-Adjusted Incidence That Lie Within Predictive Credible Bands **eResults 1.** The Results of Predictions **eFigure 2.** Projections of Age-Adjusted Incidence Rates of 32 Cancers in China **eTable 4.** Predicted 32 Cancer Incidence in China in 2018-2032, With Changes From Risk and Demographics Decomposed **eResults 2.** Sensitivity Analysis **eTable 5.** The Sensitivity Analysis of Predictions for 32 Cancer Incidence From 2018 to 2032 in China, by Sexes
eReferences

Supplement 2.
Data Sharing Statement

## References

[1] Bray F, Laversanne M, Weiderpass E, Soerjomataram I. The ever-increasing importance of cancer as a leading cause of premature death worldwide. Cancer. 2021;127(16):3029-3030. doi:10.1002/cncr.33587 34086348

[2] Cao W, Chen HD, Yu YW, Li N, Chen WQ. Changing profiles of cancer burden worldwide and in China: a secondary analysis of the global cancer statistics 2020. Chin Med J (Engl). 2021;134(7):783-791. doi:10.1097/CM9.0000000000001474 33734139 PMC8104205

[3] Soerjomataram I, Bray F. Planning for tomorrow: global cancer incidence and the role of prevention 2020-2070. Nat Rev Clin Oncol. 2021;18(10):663-672. doi:10.1038/s41571-021-00514-z 34079102

[4] Siegel RL, Giaquinto AN, Jemal A. Cancer statistics, 2024. CA Cancer J Clin. 2024;74(1):12-49. doi:10.3322/caac.21820 38230766

[5] Han B, Zheng R, Zeng H, . Cancer incidence and mortality in China, 2022. J Natl Cancer Cent. 2024;4(1):47-53. doi:10.1016/j.jncc.2024.01.006 39036382 PMC11256708

[6] Shi Z, Lin J, Wu Y, Fu S, Wan Y, Fang Y. Burden of cancer and changing cancer spectrum among older adults in China: trends and projections to 2030. Cancer Epidemiol. 2022;76:102068. doi:10.1016/j.canep.2021.102068 34864577

[7] Zhou J, Zheng R, Zhang S, . Gastric and esophageal cancer in China 2000 to 2030: recent trends and short-term predictions of the future burden. Cancer Med. 2022;11(8):1902-1912. doi:10.1002/cam4.4586 35148032 PMC9041080

[8] Zheng R, Qu C, Zhang S, . Liver cancer incidence and mortality in China: temporal trends and projections to 2030. Chin J Cancer Res. 2018;30(6):571-579. doi:10.21147/j.issn.1000-9604.2018.06.01 30700925 PMC6328503

[9] Lei S, Zheng R, Zhang S, . Breast cancer incidence and mortality in women in China: temporal trends and projections to 2030. Cancer Biol Med. 2021;18(3):900-909. doi:10.20892/j.issn.2095-3941.2020.0523 34002584 PMC8330522

[10] Wang Y, Yan Q, Fan C, . Overview and countermeasures of cancer burden in China. Sci China Life Sci. 2023;66(11):2515-2526. doi:10.1007/s11427-022-2240-6 37071289 PMC10111086

[11] World Bank Data. World Bank Group. Accessed February 28, 2022. http://data.worldbank.org

[12] Sun D, Li H, Cao M, . Cancer burden in China: trends, risk factors and prevention. Cancer Biol Med. 2020;17(4):879-895. doi:10.20892/j.issn.2095-3941.2020.0387 33299641 PMC7721090

[13] Parkin DM, Muir CS, Whelan SL, . Cancer Incidence in Five Continents, Vol. VI. IARC Scientific Publications No. 120; 1992. 1284606

[14] Parkin DM, Whelan SL, Ferlay J, Raymond L, Young J. Cancer Incidence in Five Continents, Vol. VII. IARC Scientific Publications No. 143; 1997.

[15] Parkin DM, Whelan SL, Ferlay J, Teppo L, Thomas DB. Cancer Incidence in Five Continents, Vol. VIII. IARC Scientific Publications No. 155; 2002.

[16] Curado MP, Edwards B, Shin HR, . Cancer Incidence in Five Continents, Vol. IX. IARC Scientific Publications No. 160; 2007.

[17] Forman D, Bray F, Brewster DH, et al. Cancer Incidence in Five Continents, Vol. X. IARC Scientific Publications No. 164; 2014.

[18] Bray F, Colombet M, Mery L, . Cancer Incidence in Five Continents, Vol XI. Accessed March 23, 2020. https://ci5.iarc.fr

[19] Bray F, Colombet JF, Aitken A, et al. Cancer Incidence in Five Continents, Vol. XII. Accessed October 13, 2023. https://ci5.iarc.who.int

[20] United Nations Department of Economic and Social Affairs Population Division. 2024 Revision of World Population Prospects. Accessed August 30, 2024. https://population.un.org/wpp/

[21] Segi M. Cancer Mortality for Selected Sites in 24 Countries. Dept of Public Health, Tohoku University of Medicine; 1960:1950-1957.

[22] Lin L, Yan L, Liu Y, Yuan F, Li H, Ni J. Incidence and death in 29 cancer groups in 2017 and trend analysis from 1990 to 2017 from the Global Burden of Disease Study. J Hematol Oncol. 2019;12(1):96. doi:10.1186/s13045-019-0783-9 31511035 PMC6740016

[23] Kim HJ, Fay MP, Feuer EJ, Midthune DN. Permutation tests for joinpoint regression with applications to cancer rates. Stat Med. 2000;19(3):335-351. doi:10.1002/(SICI)1097-0258(20000215)19:3<335::AID-SIM336>3.0.CO;2-Z 10649300

[24] Riebler A, Held L. Projecting the future burden of cancer: bayesian age-period-cohort analysis with integrated nested Laplace approximations. Biom J. 2017;59(3):531-549. doi:10.1002/bimj.201500263 28139001

[25] Schmid V, Held L. Bayesian extrapolation of space-time trends in cancer registry data. Biometrics. 2004;60(4):1034-1042. doi:10.1111/j.0006-341X.2004.00259.x 15606424

[26] Møller B, Fekjaer H, Hakulinen T, . Prediction of cancer incidence in the Nordic countries up to the year 2020. Eur J Cancer Prev. 2002;11(suppl 1):S1-S96. 12442806

[27] Li M, Pei J, Xu M, . Changing incidence and projections of thyroid cancer in mainland China, 1983-2032: evidence from Cancer Incidence in Five Continents. Cancer Causes Control. 2021;32(10):1095-1105. doi:10.1007/s10552-021-01458-6 34152517

[28] He H, Liang L, Han D, Xu F, Lyu J. Different trends in the incidence and mortality rates of prostate cancer between China and the USA: a joinpoint and age-period-cohort analysis. Front Med (Lausanne). 2022;9:824464. doi:10.3389/fmed.2022.824464 35187007 PMC8850968

[29] Bukavina L, Bensalah K, Bray F, . Epidemiology of renal cell carcinoma: 2022 update. Eur Urol. 2022;82(5):529-542. doi:10.1016/j.eururo.2022.08.019 36100483

[30] Bergengren O, Pekala KR, Matsoukas K, . 2022 Update on prostate cancer epidemiology and risk factors—a systematic review. Eur Urol. 2023;84(2):191-206. doi:10.1016/j.eururo.2023.04.021 37202314 PMC10851915

[31] Sun K, Zheng R, Lei L, . Trends in incidence rates, mortality rates, and age-period-cohort effects of cervical cancer—China, 2003-2017. China CDC Wkly. 2022;4(48):1070-1076. doi:10.46234/ccdcw2022.216 36751372 PMC9889234

[32] Fang Y, Li Z, Chen H, . Burden of lung cancer along with attributable risk factors in China from 1990 to 2019, and projections until 2030. J Cancer Res Clin Oncol. 2023;149(7):3209-3218. doi:10.1007/s00432-022-04217-5 35904601 PMC11797656

[33] Wang C, Chang Y, Ren J, . Modifiable risk-attributable and age-related burden of lung cancer in China, 1990-2019. Cancer. 2023;129(18):2871-2886. doi:10.1002/cncr.34850 37221876

[34] Hu M, Li M, Lin Y, . Age-specific incidence trends of 32 cancers in China, 1983 to 2032: evidence from Cancer Incidence in Five Continents. Int J Cancer. Published online July 8, 2024. doi:10.1002/ijc.35082 38973577

[35] Zhong L, Goldberg MS, Parent ME, Hanley JA. Exposure to environmental tobacco smoke and the risk of lung cancer: a meta-analysis. Lung Cancer. 2000;27(1):3-18. doi:10.1016/S0169-5002(99)00093-8 10672779

[36] Liu S, Chen Q, Guo L, . Incidence and mortality of lung cancer in China, 2008**-**2012. Chin J Cancer Res. 2018;30(6):580-587. doi:10.21147/j.issn.1000-9604.2018.06.02 30700926 PMC6328502

[37] Wang M, Luo X, Xu S, . Trends in smoking prevalence and implication for chronic diseases in China: serial national cross-sectional surveys from 2003 to 2013. Lancet Respir Med. 2019;7(1):35-45. doi:10.1016/S2213-2600(18)30432-6 30482646

[38] Guo Y, Zeng H, Zheng R, . The burden of lung cancer mortality attributable to fine particles in China. Sci Total Environ. 2017;579:1460-1466. doi:10.1016/j.scitotenv.2016.11.147 27913022

[39] Xu M, Li M, Pei J, . Gender disparities in incidence and projections of lung cancer in China and the United States from 1978 to 2032: an age-period-cohort analysis. Cancer Causes Control. 2022;33(10):1247-1259. doi:10.1007/s10552-022-01597-4 35916964

[40] Wu Y, Li Y, Giovannucci E. Potential impact of time trend of lifestyle risk factors on burden of major gastrointestinal cancers in China. Gastroenterology. 2021;161(6):1830-1841.e8. doi:10.1053/j.gastro.2021.08.006 34389341

[41] He S, Xia C, Li H, . Cancer profiles in China and comparisons with the USA: a comprehensive analysis in the incidence, mortality, survival, staging, and attribution to risk factors. Sci China Life Sci. 2024;67(1):122-131. doi:10.1007/s11427-023-2423-1 37755589

[42] Xia C, Dong X, Li H, . Cancer statistics in China and United States, 2022: profiles, trends, and determinants. Chin Med J (Engl). 2022;135(5):584-590. doi:10.1097/CM9.0000000000002108 35143424 PMC8920425

[43] Qiu H, Cao S, Xu R. Cancer incidence, mortality, and burden in China: a time-trend analysis and comparison with the United States and United Kingdom based on the global epidemiological data released in 2020. Cancer Commun (Lond). 2021;41(10):1037-1048. doi:10.1002/cac2.12197 34288593 PMC8504144

[44] Li M, Sun Y, Yang J, . Time trends and other sources of variation in *Helicobacter pylori* infection in mainland China: a systematic review and meta-analysis. Helicobacter. 2020;25(5):e12729. doi:10.1111/hel.12729 32686261

[45] Sun Z, Chen T, Thorgeirsson SS, . Dramatic reduction of liver cancer incidence in young adults: 28 year follow-up of etiological interventions in an endemic area of China. Carcinogenesis. 2013;34(8):1800-1805. doi:10.1093/carcin/bgt007 23322152 PMC3731800

[46] Liu Z, Yang Q, Shi O, Ye W, Chen X, Zhang T. The epidemiology of hepatitis B and hepatitis C infections in China from 2004 to 2014: an observational population-based study. J Viral Hepat. 2018;25(12):1543-1554. doi:10.1111/jvh.12938 29851287

[47] Tsoi KK, Hirai HW, Chan FC, Griffiths S, Sung JJ. Cancer burden with ageing population in urban regions in China: projection on cancer registry data from World Health Organization. Br Med Bull. 2017;121(1):83-94. doi:10.1093/bmb/ldw050 27913398

[48] Bray F, Jemal A, Torre LA, Forman D, Vineis P. Long-term realism and cost-effectiveness: primary prevention in combatting cancer and associated inequalities worldwide. J Natl Cancer Inst. 2015;107(12):djv273. doi:10.1093/jnci/djv273 26424777 PMC4673394

[49] Zhang M, Zhong Y, Zhao Z, . Cervical cancer screening rates among Chinese women—China, 2015. China CDC Wkly. 2020;2(26):481-486. doi:10.46234/ccdcw2020.128 34594684 PMC8393124

[50] Zhang M, Zhong Y, Bao H, . Breast cancer screening rates among women aged 20 years and above— China, 2015. China CDC Wkly. 2021;3(13):267-273. doi:10.46234/ccdcw2021.078 34594864 PMC8392981

[51] Bray F, Loos AH, McCarron P, . Trends in cervical squamous cell carcinoma incidence in 13 European countries: changing risk and the effects of screening. Cancer Epidemiol Biomarkers Prev. 2005;14(3):677-686. doi:10.1158/1055-9965.EPI-04-0569 15767349

[52] Holme Ø, Løberg M, Kalager M, . Effect of flexible sigmoidoscopy screening on colorectal cancer incidence and mortality: a randomized clinical trial. JAMA. 2014;312(6):606-615. doi:10.1001/jama.2014.8266 25117129 PMC4495882

[53] Wong LP, Han L, Li H, Zhao J, Zhao Q, Zimet GD. Current issues facing the introduction of *Human papillomavirus* vaccine in China and future prospects. Hum Vaccin Immunother. 2019;15(7-8):1533-1540. doi:10.1080/21645515.2019.1611157 31017500 PMC6746483

