# Supplementary material for: Trends in Cancer Incidence and Potential Associated Factors in China
Source: JAMA Netw Open. 2024 Oct 21;7(10):e2440381. doi: 10.1001/jamanetworkopen.2024.40381 (PMC11581522; doi:10.1001/jamanetworkopen.2024.40381)
Supplement: Supplement 1. — eMethods. Construction of BAPC Models of 32 Cancer Types eTable 1. Established BAPC Models for 32 Cancer Types by Sexes eFigure 1. Retrospective Projections of Age-Adjusted Incidence Rates of 32 Cancer Types in China for Males and Females eTable 2. The Bias and Precision of the BAPC Model for 32 Cancer Types by Sexes eTable 3. The Percentage (%) of Observed Age-Adjusted Incidence That Lie Within Predictive Credible Bands eResults 1. The Results of Predictions eFigure 2. Projections of Age-Adjusted Incidence Rates of 32 Cancers in China eTable 4. Predicted 32 Cancer Incidence in China in 2018-2032, With Changes From Risk and Demographics Decomposed eResults 2. Sensitivity Analysis eTable 5. The Sensitivity Analysis of Predictions for 32 Cancer Incidence From 2018 to 2032 in China, by Sexes eReferences [file jamanetwopen-e2440381-s001.pdf]

Supplementary Online Content

Li M, Hu M, Jiang L, Pei J, Zhu C. Trends in cancer incidence and the potential associated factors in China. *JAMA Netw Open*. 2024;7(10):e2440381. doi:10.1001/jamanetworkopen.2024.40381

eMethods. Construction of BAPC Models of 32 Cancer Types

eTable 1. Established BAPC Models for 32 Cancer Types by Sexes

eFigure 1. Retrospective Projections of Age-Adjusted Incidence Rates of 32 Cancer Types in China for Males and Females

eTable 2. The Bias and Precision of the BAPC Model for 32 Cancer Types by Sexes

eTable 3. The Percentage (%) of Observed Age-Adjusted Incidence That Lie Within Predictive Credible Bands

eResults 1. The Results of Predictions

eFigure 2. Projections of Age-Adjusted Incidence Rates of 32 Cancers in China

eTable 4. Predicted 32 Cancer Incidence in China in 2018-2032, With Changes From Risk and Demographics Decomposed

eResults 2. Sensitivity Analysis

eTable 5. The Sensitivity Analysis of Predictions for 32 Cancer Incidence From 2018 to 2032 in China, by Sexes

eReferences

© 2024 Li M et al. *JAMA Network Open.*

eMethods: Construction of BAPC models of 32 cancer types

In this study, we determined the final model based on the predictive performance of a retrospective prediction of incidence in 2013-2017. The performance was evaluated using three metrics: precision, coverage, and bias. Precision was measured by posterior standard deviation (Sd).[1] Coverage was calculated as the percentages of observed values that lie within some predictive credible bands.[1-3] Bias compared the expected incidence (*E*) generated by the projection models to the observed incidence (*O*) in 2013-2017, which was computed as absolute percentage error (APE):

$$\mathrm{APE} = \frac{|E-O|}{O} \times 100.$$ [1,4]

The final decision of models was shown in eTable 1, and the predictive performance of models were displayed in eTable 2 and eTable 3. eFigure 1 displays the results of the retrospective projections.

© 2024 Li M et al. *JAMA Network Open.*

eTable 1 Established BAPC models for 32 cancer types by sexes.

	Priors	
	Male	Female
C00-14, Oral cavity and pharynx	P1C2	P1C2
C15, Oesophagus	P2C2	P1C1
C16, Stomach	P2C2	P2C2
C17, Small intestine	P2C2	P1C2
C18-21, Colorectum	P2C2	P2C2
C22, Liver	P2C2	P2C2
C23-24, Gallbladder etc.	P2C2	P1C2
C25, Pancreas	P2C2	P2C2
C30-31, Nose, sinuses etc.	P2C2	P2C2
C32, Larynx	P2C2	P1C2
C33-34, Lung (incl. trachea and bronchus)	P2C2	P2C2
C40-41, Bone	P2C2	P2C2
C43, Melanoma of skin	P2C2	P2C2
C44, Other skin	P1C2	P1C2
C47+C49, Connective tissue	P1C1	P1C1
C50, Breast	P1C2	P2C2
C53, Cervix uteri	P1C1	P1C1
C54, Corpus uteri	P1C2	P1C2
C56, Ovary	P2C2	P2C2
C61, Prostate	P2C2	P2C2
C62, Testis	P1C2	P1C2
C60, Penis	P2C2	P2C2
C67, Bladder	P2C2	P2C2
C64-66,C68, Kidney etc.	P1C2	P1C2
C69, Eye	P2C2	P2C2
C70-72, Brain, central nervous system	P2C2	P2C2

© 2024 Li M et al. *JAMA Network Open.*

C73, Thyroid	P2C2	P2C2
C74-75, Other endocrine	P1C1	P1C1
C81, Hodgkin lymphoma	P2C2	P2C2
C82-86,C96, Non-Hodgkin lymphoma	P2C2	P2C2
C88,C90, Multiple myeloma	P2C2	P2C2
C91-95, Leukaemia	P2C2	P2C2

P: Prior for Period; C: Prior for Cohort; 1: RW1; 2:RW2. Eg: P1C2 means we set RW1 for Period effect and RW2 for Cohort effect. The priors of the age effects were set RW2 for all cancers.

© 2024 Li M et al. *JAMA Network Open.*

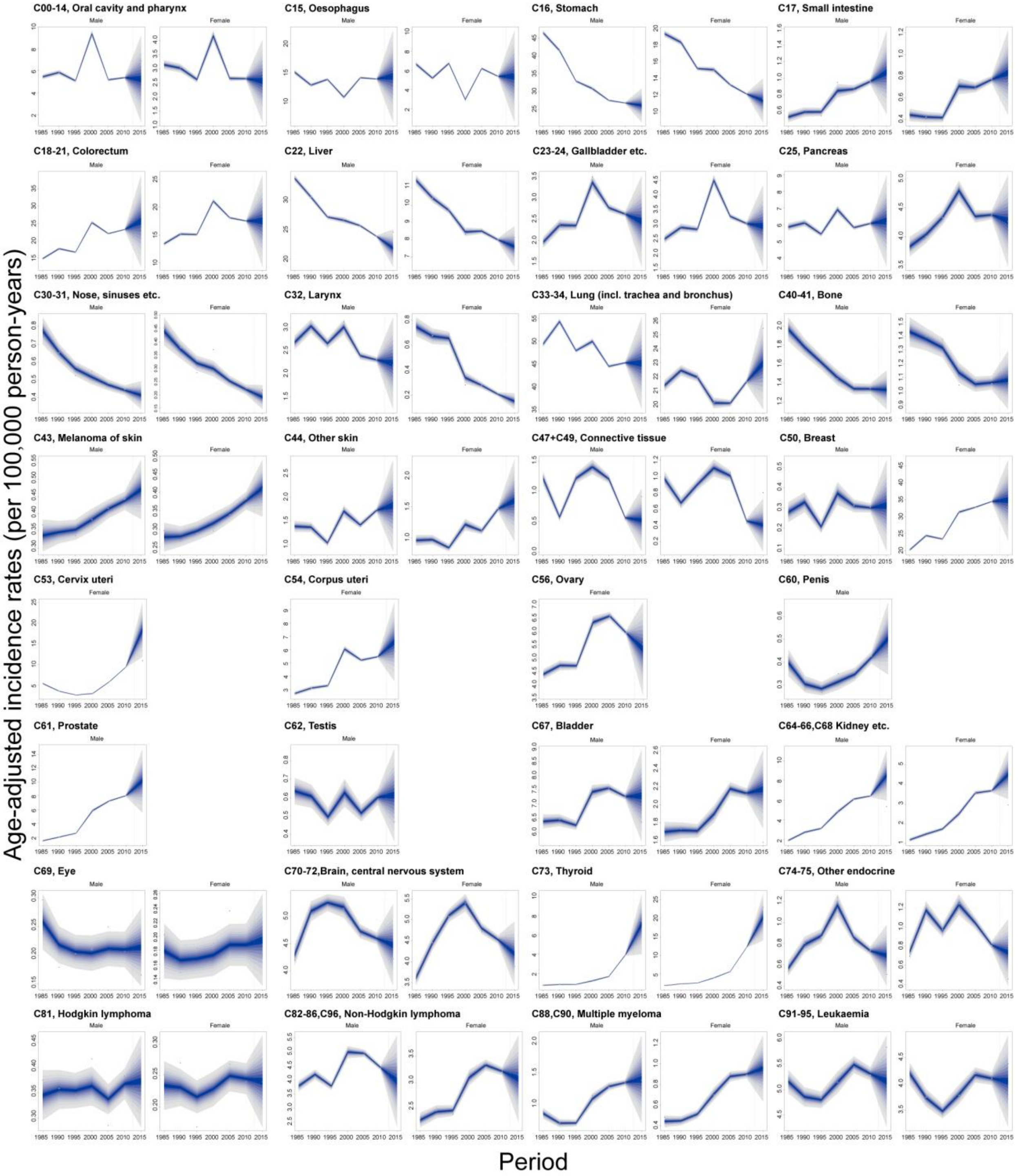

eFigure 1 Retrospective projections of age-adjusted incidence rates of 32 cancer types in China for males and females.

Fan represents 5%-95% quantiles; solid line shows predictive mean; circle represents observed incidence.

© 2024 Li M et al. *JAMA Network Open.*

eTable 2 The bias and precision of the BAPC model for 32 cancer types by sexes

	APE (%)		Sd (100,000)	
	Male	Female	Male	Female
C00-14, Oral cavity and pharynx	24.22	13.76	2.40	0.88
C15, Oesophagus	10.29	5.14	4.83	2.90
C16, Stomach	3.81	1.37	2.91	1.60
C17, Small intestine	5.08	13.77	0.31	0.24
C18-21, Colorectum	7.75	6.99	8.02	5.01
C22, Liver	11.00	6.36	1.92	0.71
C23-24, Gallbladder etc.	5.50	6.48	0.65	0.85
C25, Pancreas	1.97	0.20	1.69	0.49
C30-31, Nose, sinuses etc.	7.12	12.17	0.05	0.03
C32, Larynx	2.16	13.83	0.61	0.04
C33-34, Lung (incl. trachea and bronchus)	8.93	9.77	6.14	2.00
C40-41, Bone	3.94	2.28	0.13	0.13
C43, Melanoma of skin	9.87	0.88	0.05	0.05
C44, Other skin	6.70	7.84	0.59	0.40
C47+C49, Connective tissue	42.79	49.44	0.31	0.20
C50, Breast	9.23	6.31	0.14	7.26
C53, Cervix uteri	NA	68.63	NA	4.00
C54, Corpus uteri	NA	12.44	NA	1.81
C56, Ovary	NA	3.06	NA	1.06
C61, Prostate	11.64	NA	2.82	NA
C62, Testis	31.15	NA	0.15	NA
C60, Penis	6.66	NA	0.10	NA
C67, Bladder	14.52	37.34	0.99	0.27
C64-66,C68, Kidney etc.	58.44	52.26	1.55	0.76
C69, Eye	28.89	12.86	0.04	0.04

© 2024 Li M et al. *JAMA Network Open.*

C70-72, Brain, central nervous system	6.17	7.80	0.46	0.47
C73, Thyroid	5.02	11.77	1.89	3.77
C74-75, Other endocrine	34.97	60.17	0.18	0.21
C81, Hodgkin lymphoma	3.65	0.24	0.06	0.04
C82-86,C96, Non-Hodgkin lymphoma	11.46	2.01	1.00	0.51
C88,C90, Multiple myeloma	1.03	2.84	0.43	0.19
C91-95, Leukaemia	11.40	7.58	0.55	0.53

© 2024 Li M et al. *JAMA Network Open.*

eTable 3 The percentage (%) of observed age-adjusted incidence that lie within predictive credible bands.

	Credibility level (%)				
	**10**	**30**	**50**	**80**	**95**
Female	20.69	34.48	68.97	82.76	89.66
Male	10.34	31.03	62.07	93.10	96.55

© 2024 Li M et al. *JAMA Network Open.*

eResults: The results of predictions

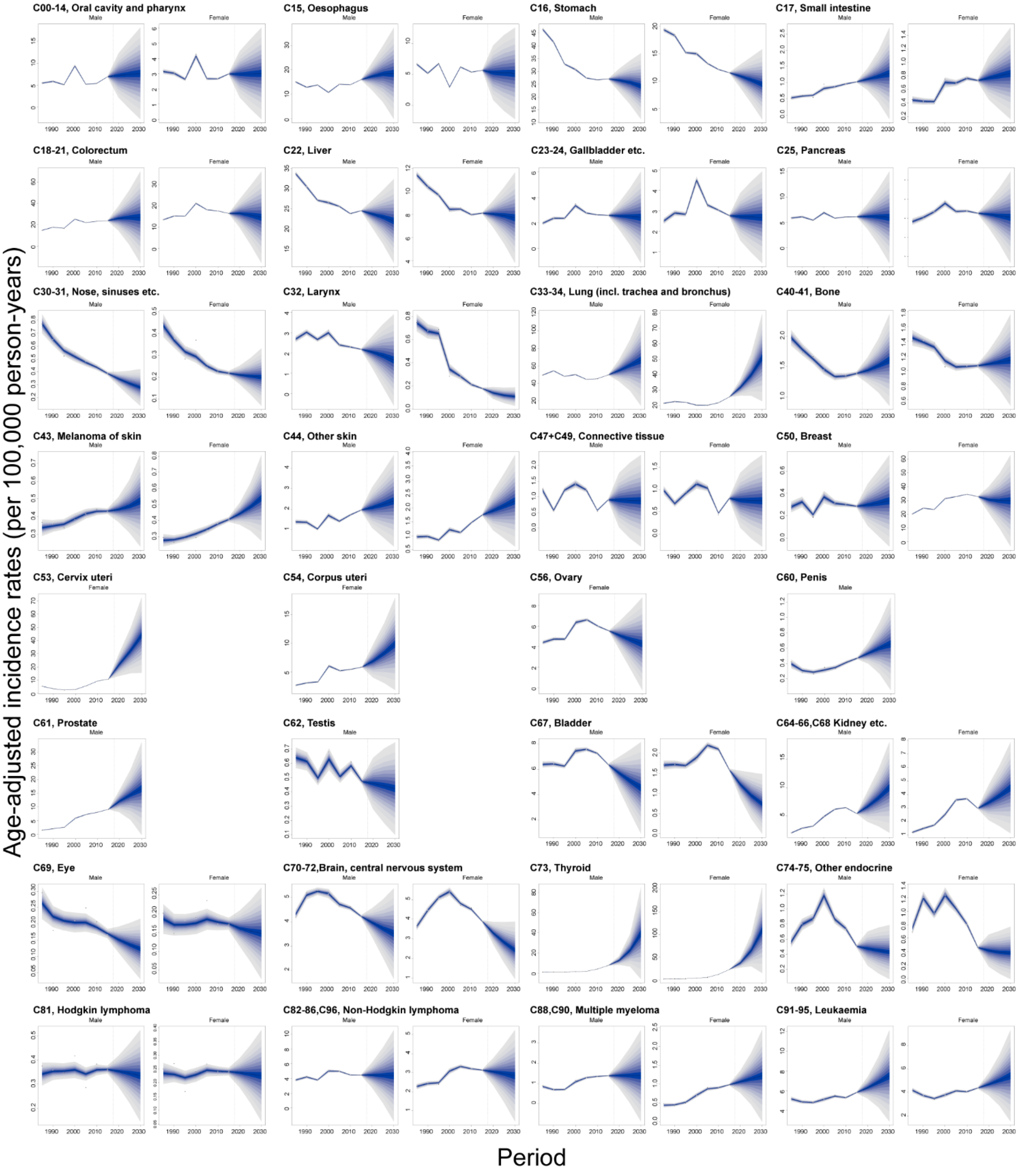

eFigure 2 Projections of age-adjusted incidence rates of 32 cancers in China.

Fan represents 5%-95% quantiles; solid line shows predictive mean; circle represents observed incidence.

© 2024 Li M et al. *JAMA Network Open.*

eTable 4 Predicted 32 cancer incidence in China in 2018-2032, with changes[b] from risk and demographics decomposed

Cancer	Period	Male							Female						
		AIR (100000)	No. of new cases	Total change(%)	Change(%) due to[c]				AIR (100000)	No. of new cases	Total change(%)	Change(%) due to[c]			
					risk	pop	age	size				risk	pop	age	size
C00-14, Oral cavity and pharynx	2018-2022	7.20	417312	28.87	17.40	11.47	9.51	1.96	2.96	170632	17.03	5.08	11.94	9.63	2.31
	2023-2027	7.44	539534	66.61	44.32	22.29	20.53	1.76	2.97	201154	37.96	13.86	24.10	21.44	2.66
	2028-2032	7.66	710525	119.41	87.27	32.15	31.28	0.87	3.01	238354	63.47	27.40	36.07	33.76	2.31
C15, Oesophagus	2018-2022	17.33	992212	30.75	12.92	17.83	15.87	1.96	5.27	398158	28.01	7.44	20.56	18.25	2.31
	2023-2027	18.33	1315786	73.39	35.26	38.13	36.37	1.76	5.23	543032	74.58	29.31	45.27	42.61	2.66
	2028-2032	18.55	1863855	145.61	86.27	59.34	58.47	0.87	5.15	731858	135.29	62.46	72.82	70.52	2.31
C16, Stomach	2018-2022	26.53	1483110	15.30	-1.85	17.16	15.20	1.96	10.91	680001	11.14	-6.18	17.32	15.01	2.31
	2023-2027	25.70	1719563	33.69	-3.23	36.92	35.16	1.76	10.20	767217	25.40	-11.45	36.85	34.19	2.66
	2028-2032	24.22	1971902	53.31	-4.74	58.05	57.18	0.87	9.42	878535	43.59	-14.56	58.15	55.84	2.31

© 2024 Li M et al. *JAMA Network Open.*

Cancer	Period	Male							Female						
		AIR (100000)	No. of new cases	Total change(%)	Change(%) due to[c]				AIR (100000)	No. of new cases	Total change(%)	Change(%) due to[c]			
					risk	pop	age	size				risk	pop	age	size
C17, Small intestine	2018-2022	1.10	61527	26.93	11.71	15.22	13.26	1.96	0.77	48476	27.31	10.46	16.85	14.53	2.31
	2023-2027	1.19	81361	67.84	35.85	32.00	30.24	1.76	0.80	61227	60.80	25.43	35.37	32.71	2.66
	2028-2032	1.28	121506	150.66	100.62	50.04	49.17	0.87	0.84	76905	101.98	47.52	54.46	52.15	2.31
C18-21, Colorectum	2018-2022	25.98	1506084	32.62	16.53	16.09	14.14	1.96	16.45	1074387	23.31	6.13	17.17	14.86	2.31
	2023-2027	26.75	2108973	85.71	51.29	34.43	32.67	1.76	15.61	1370022	57.23	20.55	36.68	34.03	2.66
	2028-2032	27.00	3637148	220.28	166.01	54.27	53.40	0.87	14.59	2048227	135.07	77.29	57.78	55.47	2.31
C22, Liver	2018-2022	23.72	1288153	10.34	-2.79	13.13	11.18	1.96	8.05	505795	17.13	-0.18	17.31	14.99	2.31
	2023-2027	22.98	1433275	22.77	-3.83	26.60	24.85	1.76	7.92	596286	38.08	1.07	37.01	34.35	2.66
	2028-2032	21.97	1589469	36.15	-3.82	39.98	39.11	0.87	7.73	704258	63.08	4.35	58.73	56.43	2.31
C23-24, Gallbladder etc.	2018-2022	2.54	148783	19.86	2.29	17.56	15.60	1.96	2.72	185451	19.98	1.07	18.91	16.60	2.31

© 2024 Li M et al. *JAMA Network Open.*

Cancer	Period	Male							Female						
		AIR (100000)	No. of new cases	Total change(%)	Change(%) due to[c]				AIR (100000)	No. of new cases	Total change(%)	Change(%) due to[c]			
					risk	pop	age	size				risk	pop	age	size
	2023-2027	2.50	196282	58.12	19.91	38.21	36.45	1.76	2.70	227462	47.16	5.48	41.68	39.03	2.66
	2028-2032	2.44	315684	154.31	92.29	62.02	61.15	0.87	2.68	282851	83.00	14.77	68.23	65.92	2.31
C25, Pancreas	2018-2022	6.14	353766	21.67	4.55	17.11	15.16	1.96	4.20	278566	17.90	-1.42	19.32	17.01	2.31
	2023-2027	6.10	471238	62.07	24.84	37.23	35.47	1.76	4.12	331633	40.36	-2.05	42.41	39.75	2.66
	2028-2032	5.98	747664	157.14	98.00	59.14	58.27	0.87	4.05	400637	69.56	0.97	68.59	66.29	2.31
C30-31, Nose, sinuses etc.	2018-2022	0.35	18045	1.45	-11.43	12.87	10.92	1.96	0.21	11699	8.51	-4.01	12.52	10.21	2.31
	2023-2027	0.31	18217	2.41	-23.45	25.86	24.10	1.76	0.20	12958	20.19	-5.55	25.74	23.08	2.66
	2028-2032	0.28	18449	3.71	-34.11	37.82	36.95	0.87	0.20	14729	36.62	-3.11	39.73	37.43	2.31
C32, Larynx	2018-2022	2.05	115896	12.05	-4.62	16.67	14.71	1.96	0.14	8793	-7.28	-25.31	18.03	15.72	2.31
	2023-2027	1.88	136997	32.45	-2.37	34.81	33.05	1.76	0.12	8540	-9.94	-49.51	39.57	36.91	2.66

© 2024 Li M et al. *JAMA Network Open.*

Cancer	Period	Male							Female						
		AIR (100000)	No. of new cases	Total change(%)	Change(%) due to[c]				AIR (100000)	No. of new cases	Total change(%)	Change(%) due to[c]			
					risk	pop	age	size				risk	pop	age	size
	2028-2032	1.66	182567	76.50	24.29	52.21	51.34	0.87	0.11	8653	-8.75	-71.63	62.88	60.58	2.31
C33-34, Lung (incl. trachea and bronchus)	2018-2022	54.70	3083022	30.49	13.00	17.48	15.53	1.96	31.56	1971492	46.37	29.20	17.16	14.85	2.31
	2023-2027	60.65	4144175	75.40	37.48	37.92	36.16	1.76	39.75	2896300	115.03	78.47	36.55	33.89	2.66
	2028-2032	66.65	5734837	142.73	82.93	59.79	58.93	0.87	50.85	4328410	221.35	164.39	56.96	54.65	2.31
C40-41, Bone	2018-2022	1.43	66928	14.89	3.81	11.09	9.13	1.96	1.09	53674	14.56	1.94	12.62	10.31	2.31
	2023-2027	1.51	77958	33.83	10.18	23.65	21.89	1.76	1.11	62279	32.92	6.69	26.23	23.57	2.66
	2028-2032	1.60	91408	56.92	19.94	36.98	36.11	0.87	1.14	72639	55.04	15.44	39.60	37.29	2.31
C43, Melanoma of skin	2018-2022	0.44	23074	15.95	2.33	13.63	11.67	1.96	0.44	25007	24.09	9.47	14.62	12.31	2.31
	2023-2027	0.46	26967	35.52	6.16	29.36	27.60	1.76	0.49	30991	53.79	23.85	29.93	27.28	2.66
	2028-2032	0.49	32133	61.48	14.63	46.85	45.98	0.87	0.54	38635	91.72	45.57	46.15	43.84	2.31

© 2024 Li M et al. *JAMA Network Open.*

Cancer	Period	Male							Female						
		AIR (100000)	No. of new cases	Total change(%)	Change(%) due to[c]				AIR (100000)	No. of new cases	Total change(%)	Change(%) due to[c]			
					risk	pop	age	size				risk	pop	age	size
C44, Other skin	2018-2022	2.08	118211	27.70	11.56	16.13	14.18	1.96	1.90	126511	31.76	13.96	17.80	15.49	2.31
	2023-2027	2.23	154650	67.06	31.81	35.25	33.49	1.76	2.07	167602	74.56	35.37	39.19	36.53	2.66
	2028-2032	2.41	205359	121.84	64.87	56.97	56.10	0.87	2.25	223253	132.52	68.56	63.97	61.66	2.31
C47+C49, Connective tissue	2018-2022	0.90	53225	36.35	26.58	9.77	7.81	1.96	0.76	46256	35.52	26.28	9.24	6.93	2.31
	2023-2027	0.89	70053	79.46	60.00	19.46	17.70	1.76	0.75	57290	67.84	49.95	17.89	15.23	2.66
	2028-2032	0.88	93375	139.21	108.76	30.45	29.58	0.87	0.75	71141	108.42	81.30	27.12	24.82	2.31
C50, Breast	2018-2022	0.30	17344	27.50	14.23	13.27	11.32	1.96	30.49	1660134	4.11	-5.10	9.21	6.90	2.31
	2023-2027	0.31	22734	67.13	38.95	28.18	26.42	1.76	29.70	1846884	15.82	-1.04	16.86	14.20	2.66
	2028-2032	0.33	30619	125.09	81.50	43.59	42.72	0.87	29.74	2253094	41.30	18.36	22.93	20.63	2.31

© 2024 Li M et al. *JAMA Network Open.*

Cancer	Period	Male							Female						
		AIR (100000)	No. of new cases	Total change(%)	Change(%) due to[c]				AIR (100000)	No. of new cases	Total change(%)	Change(%) due to[c]			
					risk	pop	age	size				risk	pop	age	size
C53, Cervix uteri	2018-2022	NA	NA	NA	NA	NA	NA	NA	22.22	1231011	135.08	127.57	7.51	5.20	2.31
	2023-2027	NA	NA	NA	NA	NA	NA	NA	32.42	1961777	274.64	262.18	12.45	9.80	2.66
	2028-2032	NA	NA	NA	NA	NA	NA	NA	44.13	2902904	454.36	438.75	15.61	13.31	2.31
C54, Corpus uteri	2018-2022	NA	NA	NA	NA	NA	NA	NA	6.89	390105	36.53	24.81	11.72	9.41	2.31
	2023-2027	NA	NA	NA	NA	NA	NA	NA	8.27	523987	83.39	63.51	19.87	17.21	2.66
	2028-2032	NA	NA	NA	NA	NA	NA	NA	9.89	684615	139.60	114.88	24.72	22.42	2.31
C56, Ovary	2018-2022	NA	NA	NA	NA	NA	NA	NA	5.05	269623	3.35	-6.77	10.12	7.80	2.31
	2023-2027	NA	NA	NA	NA	NA	NA	NA	4.64	285921	9.60	-9.20	18.80	16.14	2.66
	2028-2032	NA	NA	NA	NA	NA	NA	NA	4.27	323715	24.08	-2.15	26.23	23.93	2.31
C60, Penis	2018-2022	0.54	30047	34.00	19.09	14.91	12.95	1.96	NA	NA	NA	NA	NA	NA	
	2023-2027	0.60	41259	84.00	52.58	31.42	29.66	1.76	NA	NA	NA	NA	NA	NA	

© 2024 Li M et al. *JAMA Network Open.*

Cancer	Period	Male							Female						
		AIR (100000)	No. of new cases	Total change(%)	Change(%) due to[c]				AIR (100000)	No. of new cases	Total change(%)	Change(%) due to[c]			
					risk	pop	age	size				risk	pop	age	size
	2028-2032	0.66	59074	163.45	113.60	49.85	48.98	0.87	NA	NA	NA	NA	NA	NA	
C61, Prostate	2018-2022	12.06	733658	66.04	46.23	19.81	17.86	1.96	NA	NA	NA	NA	NA	NA	
	2023-2027	14.38	1140454	158.11	112.38	45.73	43.98	1.76	NA	NA	NA	NA	NA	NA	
	2028-2032	16.64	1911839	332.69	255.21	77.48	76.61	0.87	NA	NA	NA	NA	NA	NA	
C62, Testis	2018-2022	0.46	19726	3.76	1.85	1.91	-0.05	1.96	NA	NA	NA	NA	NA	NA	
	2023-2027	0.44	19912	4.74	3.36	1.38	-0.38	1.76	NA	NA	NA	NA	NA	NA	
	2028-2032	0.42	20137	5.92	3.57	2.35	1.48	0.87	NA	NA	NA	NA	NA	NA	
C64-66,C68, Kidney etc.	2018-2022	6.63	365603	45.31	31.29	14.02	12.07	1.96	3.40	203697	37.19	21.47	15.72	13.40	2.31
	2023-2027	8.16	528332	109.99	81.51	28.48	26.72	1.76	3.90	277839	87.12	54.76	32.36	29.71	2.66
	2028-2032	10.03	760100	202.11	159.19	42.92	42.05	0.87	4.49	377426	154.19	104.52	49.67	47.37	2.31

© 2024 Li M et al. *JAMA Network Open.*

Cancer	Period	Male							Female						
		AIR (100000)	No. of new cases	Total change(%)	Change(%) due to[c]				AIR (100000)	No. of new cases	Total change(%)	Change(%) due to[c]			
					risk	pop	age	size				risk	pop	age	size
C67, Bladder	2018-2022	5.70	320754	5.30	-11.85	17.15	15.19	1.96	1.23	80546	-7.80	-26.12	18.32	16.01	2.31
	2023-2027	5.08	348093	14.28	-23.49	37.77	36.01	1.76	0.95	77306	-11.51	-52.04	40.53	37.87	2.66
	2028-2032	4.53	393252	29.10	-32.20	61.30	60.43	0.87	0.74	79189	-9.35	-75.49	66.13	63.83	2.31
C69, Eye	2018-2022	0.15	4997	-4.29	-8.76	4.47	2.52	1.96	0.16	5117	-1.07	-5.91	4.84	2.52	2.31
	2023-2027	0.14	4456	-14.65	-18.48	3.82	2.06	1.76	0.15	4761	-7.95	-12.65	4.70	2.04	2.66
	2028-2032	0.12	4347	-16.74	-27.90	11.16	10.29	0.87	0.15	4911	-5.06	-18.70	13.63	11.33	2.31
C70-72, Brain, central nervous system	2018-2022	3.97	186103	3.09	-7.09	10.18	8.23	1.96	3.30	168582	-4.80	-16.97	12.16	9.85	2.31
	2023-2027	3.72	190514	5.54	-14.38	19.92	18.16	1.76	2.82	161689	-8.70	-32.67	23.98	21.32	2.66
	2028-2032	3.51	197098	9.18	-20.79	29.97	29.10	0.87	2.42	157158	-11.25	-46.55	35.29	32.99	2.31
C73, Thyroid	2018-2022	12.44	627116	85.56	81.07	4.50	2.54	1.96	37.13	1825040	77.09	71.99	5.10	2.78	2.31

© 2024 Li M et al. *JAMA Network Open.*

Cancer	Period	Male							Female						
		AIR (100000)	No. of new cases	Total change(%)	Change(%) due to[c]				AIR (100000)	No. of new cases	Total change(%)	Change(%) due to[c]			
					risk	pop	age	size				risk	pop	age	size
	2023-2027	22.49	1277904	278.13	271.83	6.30	4.54	1.76	64.66	3467881	236.50	229.62	6.89	4.23	2.66
	2028-2032	39.42	2777124	721.76	716.12	5.63	4.76	0.87	109.35	6682558	548.43	542.73	5.70	3.39	2.31
C74-75, Other endocrine	2018-2022	0.48	21667	4.40	-4.79	9.19	7.24	1.96	0.41	19815	1.60	-7.10	8.70	6.39	2.31
	2023-2027	0.45	22262	7.27	-10.39	17.65	15.89	1.76	0.39	19932	2.20	-13.32	15.52	12.86	2.66
	2028-2032	0.43	22683	9.29	-16.92	26.22	25.35	0.87	0.38	20446	4.84	-17.11	21.95	19.64	2.31
C81, Hodgkin lymphoma	2018-2022	0.35	16001	8.09	0.90	7.20	5.24	1.96	0.24	9961	5.39	-0.21	5.60	3.29	2.31
	2023-2027	0.35	17400	17.55	3.17	14.38	12.62	1.76	0.23	10622	12.40	1.16	11.24	8.58	2.66
	2028-2032	0.33	19229	29.91	7.39	22.52	21.65	0.87	0.23	11804	24.90	6.86	18.04	15.73	2.31
C82-86,C96, Non-Hodgkin lymphoma	2018-2022	4.44	235206	16.81	3.74	13.07	11.11	1.96	3.05	173992	13.66	-0.67	14.33	12.02	2.31

© 2024 Li M et al. *JAMA Network Open.*

Cancer	Period	Male							Female						
		AIR (100000)	No. of new cases	Total change(%)	Change(%) due to[c]				AIR (100000)	No. of new cases	Total change(%)	Change(%) due to[c]			
					risk	pop	age	size				risk	pop	age	size
	2023-2027	4.41	297755	47.87	20.68	27.19	25.43	1.76	2.96	202157	32.06	2.38	29.68	27.02	2.66
	2028-2032	4.35	441441	119.23	77.27	41.96	41.09	0.87	2.84	243483	59.05	14.20	44.86	42.55	2.31
C88,C90, Multiple myeloma	2018-2022	1.39	80008	22.59	5.98	16.61	14.66	1.96	1.07	65225	29.12	11.32	17.79	15.48	2.31
	2023-2027	1.39	108655	66.48	30.94	35.54	33.78	1.76	1.14	86420	71.08	34.09	36.98	34.33	2.66
	2028-2032	1.38	184901	183.31	127.64	55.66	54.79	0.87	1.23	120152	137.85	81.51	56.35	54.04	2.31
C91-95, Leukaemia	2018-2022	6.30	263730	16.74	8.28	8.46	6.50	1.96	4.69	201526	17.03	7.54	9.49	7.18	2.31
	2023-2027	6.88	311068	37.69	21.31	16.38	14.62	1.76	5.04	238416	38.45	19.98	18.47	15.82	2.66
	2028-2032	7.52	377451	67.08	41.60	25.48	24.61	0.87	5.43	290788	68.87	40.93	27.94	25.63	2.31

[b] Changes was calculated by comparing with number of new cases estimated in 2013-2017.

[c] Risk: represents changes due to risk factor; pop: represents changes due to demographics; age: represents changes due to ageing population; size: represents changes due to population size.

© 2024 Li M et al. *JAMA Network Open.*

eResults 2: Sensitivity analysis

eTable 5 The sensitivity analysis of predictions for 32 cancer incidence from 2018 to 2032 in China, by sexes

Cancer	Male			Female		
	2018-2022	2023-2027	2028-2032	2018-2022	2023-2027	2028-2032
C00-14, Oral cavity and pharynx	7.14	7.38	7.61	2.97	2.98	3.01
C15, Oesophagus	17.45	18.51	18.81	5.19	5.17	5.15
C16, Stomach	26.16	25.18	23.59	10.75	9.96	9.12
C17, Small intestine	1.11	1.20	1.31	0.77	0.81	0.84
C18-21, Colorectum	25.96	26.73	27.01	16.44	15.60	14.59
C22, Liver	23.56	22.72	21.62	8.08	7.97	7.80
C23-24, Gallbladder etc.	2.55	2.52	2.47	2.72	2.71	2.70
C25, Pancreas	6.14	6.11	5.99	4.19	4.10	4.02
C30-31, Nose, sinuses etc.	0.35	0.32	0.29	0.20	0.19	0.18
C32, Larynx	2.06	1.89	1.66	0.14	0.12	0.11
C33-34, Lung (incl. trachea and bronchus)	53.69	58.79	63.86	31.26	39.11	49.72
C40-41, Bone	1.39	1.43	1.49	1.06	1.06	1.08

© 2024 Li M et al. *JAMA Network Open.*

Cancer	Male			Female		
	2018-2022	2023-2027	2028-2032	2018-2022	2023-2027	2028-2032
C43, Melanoma of skin	0.45	0.47	0.50	0.44	0.47	0.52
C44, Other skin	2.08	2.22	2.38	1.90	2.06	2.24
C47+C49, Connective tissue	0.90	0.90	0.90	0.76	0.76	0.76
C50, Breast	0.30	0.31	0.33	30.59	29.80	29.80
C53, Cervix uteri	NA	NA	NA	22.31	32.70	44.72
C54, Corpus uteri	NA	NA	NA	6.86	8.21	9.82
C56, Ovary	NA	NA	NA	5.05	4.65	4.28
C61, Prostate	12.83	15.67	18.57	NA	NA	NA
C62, Testis	0.46	0.44	0.43	NA	NA	NA
C60, Penis	0.54	0.60	0.66	NA	NA	NA
C67, Bladder	5.70	5.10	4.55	1.23	0.95	0.74
C64-66,C68, Kidney etc.	6.59	8.08	9.89	3.39	3.90	4.48
C69, Eye	0.16	0.15	0.14	0.17	0.17	0.16
C70-72, Brain, central nervous system	3.97	3.73	3.52	3.31	2.84	2.44
C73, Thyroid	12.42	22.49	39.44	37.07	64.57	109.23

© 2024 Li M et al. *JAMA Network Open.*

Cancer	Male			Female		
	2018-2022	2023-2027	2028-2032	2018-2022	2023-2027	2028-2032
C74-75, Other endocrine	0.48	0.45	0.43	0.41	0.39	0.38
C81, Hodgkin lymphoma	0.35	0.34	0.33	0.24	0.24	0.24
C82-86,C96, Non-Hodgkin lymphoma	4.43	4.39	4.32	3.05	2.96	2.85
C88,C90, Multiple myeloma	1.39	1.39	1.38	1.06	1.14	1.23
C91-95, Leukaemia	6.27	6.80	7.38	4.68	5.02	5.38

© 2024 Li M et al. *JAMA Network Open.*

eReferences

1. Knoll M, Furkel J, Debus J, Abdollahi A, Karch A, Stock C. An R package for an integrated evaluation of statistical approaches to cancer incidence projection. BMC Med Res Methodol. 2020;20(1):257.
2. Knorr-Held L, Rainer E. Projections of lung cancer mortality in West Germany: a case study in Bayesian prediction. Biostatistics. 2001;2(1):109-29.
3. Riebler A, Held L. Projecting the future burden of cancer: Bayesian age-period-cohort analysis with integrated nested Laplace approximations. Biom J. 2017;59(3):531-49.
4. Demers A, Qiu Z, Dewar R, Shaw A. Validation of Canproj for projecting Canadian cancer incidence data. Health Promot Chronic Dis Prev Can. 2020;40(9):267-80.

© 2024 Li M et al. *JAMA Network Open.*
