# Supplementary material for: Trends in Cancer Incidence and Potential Associated Factors in China
Source: JAMA Netw Open. 2024 Oct 21;7(10):e2440381. doi: 10.1001/jamanetworkopen.2024.40381 (PMC11581522; doi:10.1001/jamanetworkopen.2024.40381)
Supplement: Supplement 2. — Data Sharing Statement [file jamanetwopen-e2440381-s002.pdf]

Data Sharing Statement

Li. Trends in Cancer Incidence and the Potential Associated Factors in China. *JAMA Netw Open*. Published October 21, 2024. doi:10.1001/jamanetworkopen.2024.40381

Data
Data available: Yes
Data types: Other (please specify)
Additional Information: Cancer incidence data (not involving human participants), Data dictionary.
How to access data: See http://ci5.iarc.fr/
When available: With publication

Supporting Documents
Document types: None

Additional Information
Who can access the data: Anyone requesting the data.
Types of analyses: For any purpose.
Mechanisms of data availability: With investigator support.
